# A real-world evaluation of the effectiveness and Sufficiency of Current Emergency Department Preventative Strategies for Reducing Emergency Department revisits in a Canadian children’s hospital: *a retrospective cohort study*

**DOI:** 10.1186/s13223-024-00900-z

**Published:** 2024-06-25

**Authors:** Tahereh Haji, Lynnette Lyzwinski, Cara Dhaliwal, Garvin Leung, Sandra Giangioppo, Dhenuka Radhakrishnan

**Affiliations:** Children’s Hospital of Eastern Ontario, Ontario, Canada

**Keywords:** Control/management, Paediatrics, Prevention, Education

## Abstract

**Background:**

Despite asthma guidelines’ recommended emergency department preventative strategies (EDPS), repeat asthma-related emergency department (ED) visits remain frequent.

**Methods:**

We performed a retrospective cohort study of children aged 1–17 years presenting with asthma to the Children’s Hospital of Eastern Ontario (CHEO) ED between September 1, 2014 – August 31, 2015. EDPS was defined as provision of education on trigger avoidance and medication technique plus documentation of an asthma action plan, a prescription for an inhaled controller medication or referral to a specialist. Logistic regression was used to identify factors associated with receipt of EDPS. We further compared the odds of repeat presentation to the ED within the following year among children who had received EDPS versus those who had not.

**Results:**

1301 patients were included, and the mean age of those who received EDPS was 5.0 years (SD = 3.7). Those with a moderate (OR = 3.67, 95% CI: 2.49, 5.52) to severe (OR = 3.69, 95% CI: 2.50, 5.45) asthma presentation were most likely to receive EDPS. Receiving EDPS did not significantly reduce the adjusted odds of repeat ED visits, (OR = 0.82, 95% CI: 0.56, 1.18, *p* = 0.28).

**Conclusions:**

Patients with higher severity asthma presentations to the ED were more likely to receive EDPS, but this did not appear to significantly decrease the proportion with a repeat asthma ED visit. These findings suggest that receipt of EDPS in the ED may not be sufficient to prevent repeat asthma ED visits in all children.

**Supplementary Information:**

The online version contains supplementary material available at 10.1186/s13223-024-00900-z.

## Introduction

Asthma is a common, chronic respiratory disease and one of the leading causes of hospitalization in children [1, 2]. It is estimated that 15% of Canadian children suffer from asthma [3], with global estimates reported to have a similar prevalence of 14% [4, 5]. Asthma exacerbations and uncontrolled asthma are associated with impaired lung function, reduced participation in physical activity, increased school absenteeism, reduced quality of life, increased use of urgent care and emergency department (ED) visits, and elevated healthcare and family expenses [6–8]. Although hospitalization rates have decreased across Canada, the re-admission rates for paediatric populations have not changed significantly over a period of ten years (2006–2016), with only a 1.1% reduction according to the Canadian Institutes for Health Information (CIHI) [9] .

Thus, it is critical to find evidence-based ways to control childhood asthma and prevent exacerbations necessitating ED visits and hospitalizations in order to improve quality of life, reduce morbidity, and decrease direct costs to the healthcare system in the long-term. The Canadian Pediatric Society (CPS) states that the essential components of ED management of asthma exacerbations include an immediate and objective assessment of the severity of the asthma exacerbation; prompt and effective medical intervention; arranging appropriate disposition of the patient after emergency management; and arranging proper follow up [2]. Appropriate disposition comprises implementation of a discharge plan that includes prescription for inhaled corticosteroids (ICS) for children with previous moderate to severe asthma exacerbations, provision of an asthma action plan (AAP), education on avoidance of asthma triggers and medication technique review, with specialist referral indicated for children with exacerbations despite prior ICS use. Similarly, the British Thoracic Society (BTS) guidelines state that discharge plans from the ED should include asthma education with an AAP, inhaler technique review, evaluation for trigger avoidance, review of the need for/optimizing preventer treatments, and arrangement of proper follow-up [10] .A summary of the updated Canadian Paediatric Society Position statement on managing an acute asthma exacerbation) [11] along with the Canadian Thoracic Society Asthma Consensus Guidelines [12] are in the appendix supplementary Table (S1).

However, appropriate disposition and follow-up arrangement can be challenging to provide in a busy ED setting [10] .Research at two hospitals in Quebec, Canada in 2005 found that only a minority (8%) of adult patients received recommended ED preventative discharge strategies (EDPS) that were in line with recommendations in the Canadian Asthma Guidelines [13]. Little is known about the prevalence of EDPS implementation in paediatric populations, particularly in Canada.

There are several risk factors linked with childhood asthma severity, ED visits and hospitalization. For example our previous research has found that a moderate to severe asthma presentation, prior diagnosis of asthma, allergy to nuts, and having a primary care provider predicted recurrent asthma ED visits [14, 15] with several additional risk factors for asthma hospitalization documented in our recent systematic review [15]. However, the extent to which these risk factors are considered at the time of arranging disposition following an asthma ED visit is not known.

Since the publishing of recent Canadian and other global guidelines for management of asthma ED visits, there has been no evaluation of compliance to these guidelines and the frequency with which these recommended EDPS are used in the ED.

Additionally, since the rate of ED revisits has not significantly changed over the last decade, there is also a need to determine whether currently recommended EDPS, and the way in which these are implemented are effective for preventing repeat ED visits in paediatric patients in the real-world setting.

In this study, we primarily aimed to determine whether recommended EDPS are effective for preventing ED re-visits in children and adolescents. We also aimed to better understand the determinants of EDPS implementation and patient level risk factors linked with a higher risk of ED re-visits. Finally, we aimed to determine the prevalence of EDPS implementation for children and adolescents in a tertiary care paediatric centre.

We hypothesized, that EDPS are used in the majority of patients in our tertiary level paediatric centre. We further hypothesized that EDPS would decrease asthma ED revisits within a 1-year time span, and that EDPS would be provided to those patients with locally determined risk factors for asthma ED re-visits: namely, those with moderate to severe ED presentations, those with allergies, and those who had a primary care provider.

## Methods

### Overview

This was a retrospective cohort study performed at a single tertiary care paediatric centre using data abstracted from each child’s medical chart and with linkage to local hospital administrative data. We observed children presenting to our centre for any asthma exacerbation for a subsequent ED re-visit within a one 1-year time period. We determined whether children had received EDPS and identified patient factors associated with the receipt of EDPS, as well as which factors were associated with ED re-visits within the following year. ED revisits are defined as visiting the emergency department (ED) revisitation within a period of one year from the last ED visit for an acute asthmatic episode (non-hospitalized cases).

### Participants and setting

We included all children aged 1–17 years of age presenting with an asthma exacerbation to the ED at the Children’s Hospital of Eastern Ontario (CHEO), between September 1, 2014 – August 31, 2015, who were discharged from the ED within that same visit. CHEO is a pediatric tertiary care center serving regions in multiple provinces, including eastern and northern Ontario, western Quebec and Nunavut, and sees over 70,000 patients in the ED annually. All patients that arrive to our ED with an asthma exacerbation are triaged based on respiratory symptom severity, as determined using the validated Pediatric Respiratory Assessment Measure (PRAM) [18]. Patients are managed based on their PRAM and per a standard pathway that conforms with recent Canadian guidelines for the management of acute asthma exacerbations [1]. On this pathway, all children with an initial PRAM of 4–12 (moderate = 4–7, severe = 8–12) are treated with systemic corticosteroids. Prior to discharge home, all patients receive education on trigger avoidance and correct inhaler technique, as per a standard checklist used by the nurses and respiratory therapists in the ED. For children with more than one ED visit during this time period, only the first visit was used as the index visit at which all baseline variables were defined.

Patients were excluded from this study if their age was less than 1 year at the time of first asthma ED presentation as the diagnosis of asthma is controversial in this age group. We also excluded patients who were already followed by an asthma specialist, defined as having had a respirology clinic visit within the 12 months prior to the index ED visit, as these patients are provided more rigorous education and medical management and would not be representative of a typical patient presenting to the ED for asthma. We excluded repeat ED visits or hospitalizations that occurred within 2 weeks of the index ED visit as our intent was to capture only new exacerbations. Immediate bounce-backs to the ED (i.e. within 2 weeks) likely represent failure of emergency management of the index asthma exacerbation and are less likely to be sensitive to EDPS against future repeat asthma ED visits, as defined in this study [15].

### Data sources

Approval was obtained from the CHEO Research Ethics Board to perform this study. Information on patient demographics, clinical characteristics, and study outcomes were extracted from each patient’s chart as well as local health administrative data at CHEO.

### Study variables

Variables collected for the index ED visit included those that we have previously shown to be associated with asthma ED visits at our centre, including demographic data (e.g., age at index ED visit treated continuously, sex, having a regular primary care provider listed in the medical chart), PRAM at triage, number of ED visits in the previous 12 months, and the presence of allergies. Allergies were categorized as those to (i) nuts (including peanuts and tree nuts), (ii) other foods (excluding nuts), or (iii) medications or inhaled allergens (e.g., pet dander, dust, pollen).

Our primary outcome was whether recommended EDPS were received by patients upon discharge from the ED. As all patients during the course of treatment in our ED already receive education on medication technique and trigger avoidance, EDPS was further defined for this study as (1) documentation of provision of a prescription for an ICS and/or (2) a printed AAP (which includes instructions for use of previously prescribed ICS) and/or (3) referral to an asthma specialist (i.e. allergist or respirologist). The delivery of EDPS was at the sole discretion of the treating ED physician [1]. Our secondary outcome was the odds of an ED re-visit within the following 12 months for children who did or did not receive EDPS. Finally, among children who received EDPS and were also referred to an asthma specialist following the index visit, we further characterized the proportion who had an ED-revisit in the following year. Asthma ED visits were defined based Ontario Health Insurance Plan diagnostic billing code 493 at discharge as documented in local health administrative data at CHEO. Asthma hospitalizations were determined by linkage with hospital health administrative data using the medical record number of each patient and identifying visits with asthma as the most responsible discharge diagnosis (i.e., International Classification of Diseases version 10 diagnostic code J45 or J46).

### Analysis

Descriptive statistics were used to compare the study groups. Individuals receiving EDPS were compared to other patients who did not receive EDPS using the t-test and the chi-square test for continuous and categorical data, respectively. Continuous data were presented as means +/- standard deviation, whereas categorical data were presented as proportions.

We constructed a multivariable logistic regression model to identify characteristics of patients who received EDPS. A second model was used to determine the patient characteristics that are associated with the odds of an asthma ED re-visit. Odds ratios were adjusted and presented with 95% confidence intervals. Statistical significance was determined at a α = 0.05. We also performed a sensitivity analysis to identify predictors of receipt of EDPS for children with PRAM ≥ 4 at triage (i.e. moderate or severe exacerbations only).

## Results

### General descriptive and patient characteristics

**Fig. 1:**
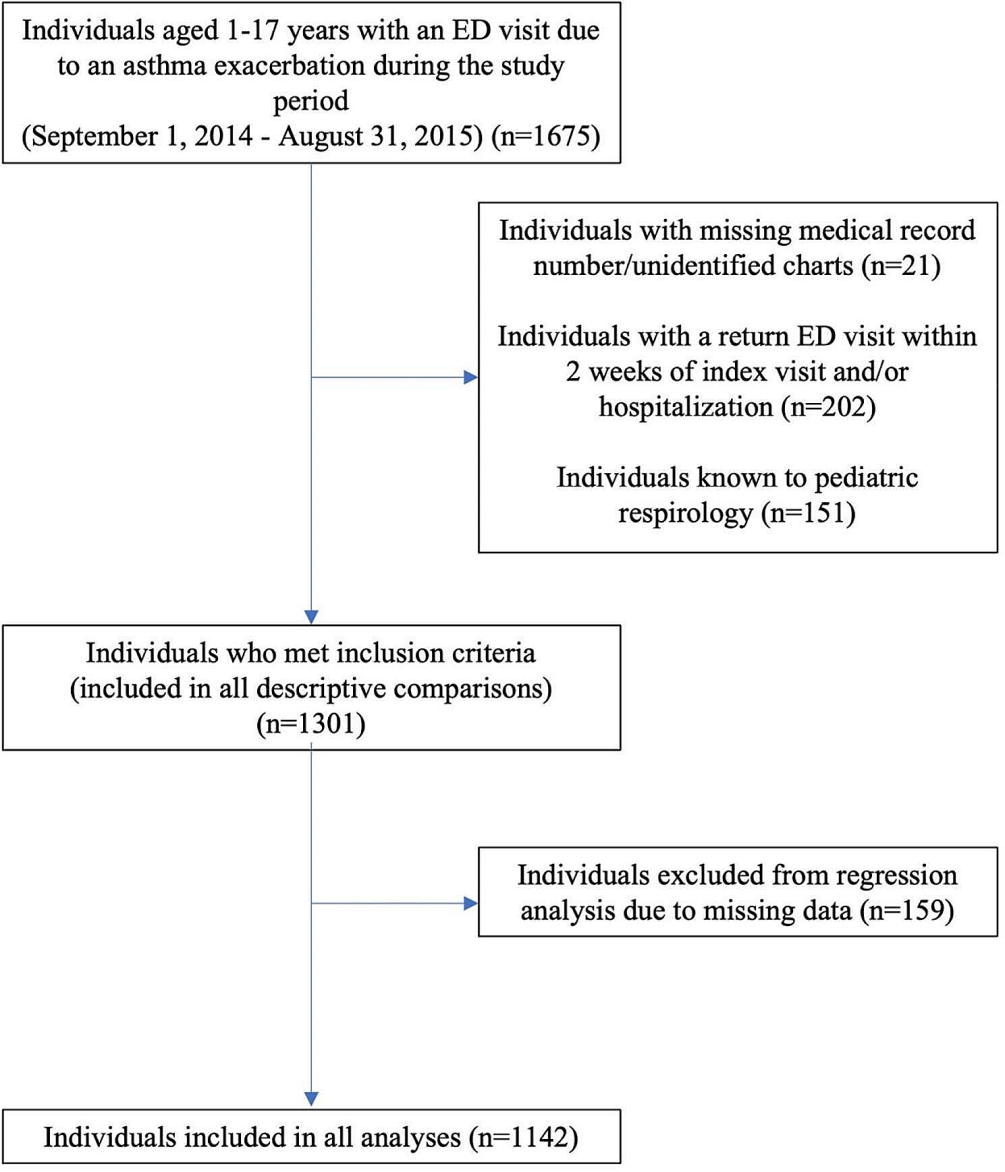
Flow diagram demonstrating cohort creation. ED = Emergency Department

Of a total of 1675 patients who had presented to the ED with an index asthma exacerbation during the study period, 1301 patients met inclusion criteria. There were 1034 patients who received EDPS (Fig. 1). The overall prevalence of EDPS implementation was 89.5%. The mean age of those who received EDPS was 5.0 (SD = 3.7) years and the mean age of those who did not receive EDPS was 6.5 (SD = 4.5).

### Emergency department prevention strategies and repeat asthma emergency department visits

A total of 290 of the study patients experienced an asthma ED re-visit (22%). Patients who received EDPS were 18% less likely to re-visit the ED in the following year, though the results were not statistically significant (OR = 0.82, 95% CI = 0.56–1.18, *P* > 0.05) in comparison to those who did not receive EDPS. Additionally, seeing a specialist was not independently associated with a lower risk of ED re-visits. A total of 32 patients were referred from the ED to an asthma specialist. Of these, 7 (20%) had a repeat asthma ED visit or hospitalization within the upcoming year, while 23% of their counterparts who did not see a specialist also had a repeat acute asthma visit (*p* = 0.64).

### Other patient level predictors of repeat asthma emergency department visits

Patients who had a prior ED visit in the past year were 1.54 more likely to have a repeat ED visit (95% CI = 1.09–2.17; p-value < 0.01) than their counterparts. Other patient level factors that were significantly associated with repeat acute asthma visits included severe PRAM at triage (OR = 1.96; 95% CI = 1.31–2.92) and having a primary care provider (OR = 2.04; 95% CI=,1.26–3.33), while age, sex, and presence of allergies were not associated with this (Table 1).

**Table 1:** Patient characteristics associated with odds of future acute asthma visits

	Adjusted Odds Ratios			
Effect	Odds Ratio	95% Confidence Interval		p-value
Age	0.96	0.92	1.00	0.07
Sex (Male vs. female)	0.94	0.70	1.26	0.69
PRAM score (moderate vs. mild)*	1.48	0.98	2.21	0.06
PRAM score (severe vs. mild)*	1.96	1.31	2.92	< 0.01
Prior diagnosis of asthma	1.61	1.10	2.37	0.02
Second hand smoke exposure	1.42	0.79	2.53	0.24
Primary care provider	2.04	1.26	3.31	< 0.01
Allergy: Nut vs. other allergies absent**	1.17	0.78	1.76	0.46
Allergy: Food vs. other allergies absent**	1.06	0.70	1.62	0.78
Allergy: Other vs. other allergies absent**	1.11	0.78	1.59	0.56
Prior ED visit within previous year	1.54	1.09	2.17	0.01
Received EDPS	0.82	0.56	1.18	0.28

PRAM = pediatric respiratory assessment measure, EDPS = emergency department preventative strategies. Definition of EDPS is having documentation of Asthma Action Plan (AAP) or controller inhaler prescribed or referral to specialist

* PRAM severity was defined as follows: Mild = 0–3; Moderate = 4–7; Severe = 8–12

***Allergies were defined as follows: Nuts = all nuts including peanuts and tree nuts; Food = all foods excluding nuts; Other = all reported allergies that were not to nuts or food types, e.g. pollen, dust, etc.

### Predictors of receiving EDPS from emergency physicians

Paediatric patients who received EDPS were 1.3 years (SD = 1.1) younger on average than their counterparts (p-value < 0.01). Additionally, a higher proportion of patients who received EDPS had moderate to severe PRAM scores on their index visit and reported sensitization to aeroallergens compared to children who did not. Among children with a moderate and severe PRAM score, 24.2% and 27.5%, respectively, did not receive EDPS. PRAM score was missing in 169 (12.2%) patients and a smaller proportion of this group received EDPS compared to those who had documented PRAM scores (65% vs. 82%) (Table 2).

**Table 2:** Characteristics of cohort at index asthma emergency department visit

	EDPS* (*n* = 1034)	EDPS not documented (*n* = 267)	*p*-value
Age, *years* mean, (SD), [95% CI]	5.0 (3.7), [4.7–5.2]	6.5 (4.5) [5.9-7.0]	< 0.01
Sex *Male* N (%)	673 (65.0%)	158 (59.1%)	0.07
PRAM at index visit			< 0.01
N (%):			
0–3 (mild) 4–7 (moderate) 8–12 (severe) Missing	176 (18.9%) 339 (36.4%) 416 (44.7%) 103 (10.0%)	102 (48.3%) 51 (24.2%) 58 (27.5%) 56 (21.0%)	
Prior history of asthma N (%)	781 (75.5%)	215 (80.5%)	0.08
**Allergy**			
N (%):			
None Nuts Food Other**	647 (62.3%) 173 (16.7%) 163 (15.8%) 211 (20.4%)	149 (55.8%) 47 (17.6%) 44 (16.5%) 77 (28.8%)	0.05 0.72 0.78 < 0.01
Second hand smoke	54 (5.2%)	13 (4.9%)	1.0
exposure N (%)			
Has a primary care physician N (%)	885 (85.6%)	235 (88.0%)	0.32
Prior ED visits N (%)	180 (17.4%)	45 (16.9%)	0.83
Return ED visit or hospitalization N (%)	233 (22.5%)	57 (21.3%)	0.74

SD = standard deviation, 95% CI = 95% confidence interval, PRAM = pediatric respiratory assessment measure, EDPS = emergency department preventative strategies

*Definition of EDPS is having documentation of Asthma Action Plan (AAP) OR controller inhaler prescribed OR referral to specialist

**Allergies were divided into categories. Nuts = all nuts including peanuts and tree nuts; food = all foods excluding nuts; other = all reported allergies that were not to nuts or food types, e.g. pollen, dust, etc.

In adjusted logistic regression analyses (limited to 1142 of the 1301 who met inclusion criteria, due to missing data for some variables), both moderate (OR = 3.67; 95% CI = 2.59–5.42) and severe PRAM (OR = 3.19; 95% CI = 2.50–5.45) at triage during the index ED visit were independently associated with a higher likelihood of receiving EDPS (p-values < 0.01). Age, sex, prior diagnosis of asthma, documented second hand smoke exposure, having a primary care provider, any allergies or prior ED visits were not significantly associated with higher odds of receiving EDPS (Table 3). Our sensitivity analysis, restricted only to patients with a moderate to severe asthma exacerbation, similarly identified the same predictors of receiving EDPS. (See Supplementary Tables S1-S3.)

**Table 3:** Patient characteristics associated with odds of receiving Emergency Department Prevention Strategies

	Adjusted Odds Ratios			
Predictor	OR	95% Confidence Interval		p-value
Age	0.97	0.93	1.01	0.10
Sex (Male vs. female)	1.08	0.78	1.49	0.64
PRAM (moderate vs. mild)*	3.67	2.49	5.42	< 0.01
PRAM (severe vs. mild)*	3.69	2.50	5.45	< 0.01
Prior diagnosis of asthma	1.05	0.68	1.62	0.82
Second hand smoke exposure	0.83	0.41	1.68	0.60
Primary care provider	0.96	0.60	1.54	0.88
Allergy: Nut vs. other allergies absent**	0.85	0.54	1.36	0.50
Allergy: Food vs. other allergies absent**	1.05	0.64	1.71	0.86
Allergy: Other vs. other allergies absent**	0.80	0.55	1.17	0.26
Prior ED visit within previous year	0.98	0.66	1.48	0.94

PRAM = pediatric respiratory assessment measure, EDPS = emergency department preventative strategies. Definition of EDPS is having documentation of Asthma Action Plan (AAP) or controller inhaler prescription or referral to asthma specialist

* PRAM severity was defined as follows: Mild = 0–3; Moderate = 4–7; Severe = 8–12

** Allergies were defined as follows: Nuts = all nuts including peanuts and tree nuts; Food = all foods excluding nuts; Other = all reported allergies that were not to nuts or food types, e.g. pollen, dust, etc.

## Discussion

In this study, our primary purpose was to gain a better understanding of the effectiveness of EDPS for managing pediatric asthma by evaluating whether it reduces the risk of repeat emergency department visits at one-year post-discharge in a real-world setting. We also sought to gain insight into the frequency of EDPS implementation at our tertiary care pediatric center. While we found that EDPS seemed to be associated with an 18% lower risk of emergency department re-visits, this association was not statistically significant. Therefore, it appears that EDPS at discharge may not be sufficient for controlling pediatric asthma and preventing repeat asthma ED visits. Although we found a high implementation of EDPS with 87% of physicians providing this to their patients and conforming to Canadian best practice guidelines for asthma management, current EDPS strategies are not enough. These recommendations should be revisited in order to reduce the observed incidence of ED re-visits in nearly 1 out of 4 patients in our study cohort.

Our findings are similar to those from a study in the United States, which likewise, did not find overall, that EDPS led to a reduction in hospital re-admission rates at 12 weeks; however, among all potential risk factors studied, strong education was considered the most essential factor to contribute to lower risk of hospital readmission in children [16]. This indicates that there is a need for stronger education and knowledge dissemination to parents and their children. Prior studies indicate that parents and caregivers desire to receive asthma management education through diverse media including videos, written material, and verbal face to face instructions with follow-up phone calls [17]. Thus, it may be that a more tailored and comprehensive approach, that could involve the diverse use of media may be needed to enhance the quality of asthma education and management provided for children and caregivers after an ED visit. As this may not always be available in the busy ED setting, increased referral to asthma specialists or educators may be needed for some patients, particularly those who may be at higher risk for ED re-visits.

Another aim of this study was to better understand the determinants of EDPS implementation and additional patient level risk factors linked with a higher risk of ED repeat visits. Given that higher PRAM scores were linked with an increased risk of ED revisits in this, and in previous studies [14, 15] stronger and targeted EDPS for this group of patients may be warranted. That is, even though we found that patients with higher PRAM scores are more likely to receive EDPS, they may benefit further from enhanced provision of these strategies as the current implementation approach is insufficient and does not result in best asthma control. The prevailing literature also supports our findings that previous ED visits increase the risk of hospital visits in the future [14, 18] yet it is important to note we did not find that patients with a previous history of ED visits were more likely to receive EDPS from their attending ED physicians. This suggests that ED physicians should be more mindful that patients with a previous history of ED visits need to be better targeted in order to optimize their receipt of EDPS. In addition to this, previous studies are also in agreement with our findings that having a primary care provider is associated with an increased risk of ED visits [19]. This may be due to confounding by indication, whereby patients with more severe asthma are more likely to seek out help from a PCP [24]. A prior study has suggested that patients with a PCP may be less likely to receive EDPS as ED physicians may assume that their PCP will manage their condition [20]. Yet up to 66% of patients do not seek follow-up care from their primary care provider [21] after an asthma ED visit, indicating that simply having a PCP is not protective against ED re-visits. Past research has found that second hand smoke significantly increases the risk of ED visits by 3.5 times and of increases the risk of hospitalization by 2.8 times in children with higher concentrations of second-hand smoke exposure based on serum cotinine measurements (> 3.0 ng/ml) relative to their counterparts, respectively [22]. However, we did not find this association in our study. This apparent lack of association in our current study may reflect the inconsistent recording of smoke exposure in a typical ED encounter and limitations of our data sources.

When considering other patient characteristics in an overall risk assessment, there are some additional risk factors that have previously been considered, including allergies and patient age. In the current study, we did not find an association between allergic history and ED re-visits, though we have previously found a higher risk for asthma hospitalizations among patients with comorbid peanut allergy at our centre, as also seen in other prior studies [23–25].This discrepancy could be accounted for by differences in study inclusion criteria; in the current study we included all children with any asthma ED visit, but excluded those who were already followed by an asthma specialist. This likely resulted in excluding a higher proportion of children with more severe asthma where a peanut allergy-association is more prominent, as evidenced by overall lower allergy prevalence in the current cohort (38.8%) compared to our previous study (62.7%). This cohort composition difference may have diminished the effect of allergies on ED re-visit risk. Future studies including larger sample sizes are needed to clarify this association.

Finally in this study, we did not find an association between young age and ED revisits; whether or not age is a potential independent patient level risk factor for ED visits is unclear per mixed findings in the literature. While some previous research has found an association between younger age and higher risk of repeat ED visits [26], other studies including ours have not found support for this [14, 19, 21, 26], indicating that age may not be as useful or of highest priority when assessing individual patient risk. Specifically, we did not find that age was a predictor of repeat asthma ED visits (OR = 0.96; p-value > 0.05; 95% CI includes 1). The study by Giangioppo et al. [14] examined 3300 paediatric ED asthma visits in Canada also did not find age to be a significant predictor of repeat asthma ED visits. As a result, we decided not to undertake further subgroup analyses stratified by age (i.e. comparing teenagers with younger children).

### Strengths and limitations

Our study has several strengths and limitations. Our chart review and data abstraction were performed directly by two of our authors, allowing us to perform a thorough search to extract relevant data. Our use of an objective diagnosis of asthma that was based on discharge diagnosis recorded in administrative data allowed reliable capture of our study population. However, we relied on ED physician documentation as our primary data source, which may not reflect true practices (i.e., in the case where a pertinent positive is documented, but a pertinent negative is not). We did encounter some missing data, such as PRAM at triage, which may have skewed our findings. In addition, our study was performed in a single paediatric tertiary care centre, and our population, has reasonably good access to health care, which may limit the generalizability of these findings to smaller community or rural EDs. Additionally, previous studies have shown that referral to a specialist improves asthma related morbidity [14], but we could not include this in our adjusted analyses due to the small percentage of patients referred to respirologists. Furthermore, we only considered variables that were readily available and documented in our ED records at the hospital, though our previous systematic review looking at predictors of future asthma hospitalization identified 28 risk-related variables, with only a small proportion included in the present study [27].

Finally, it should be noted that a major limitation of this study is that the proportion of children who did not receive EDPS is relatively small when compared with the number of children who received EDPS. However, our study was designed to be a real-life effectiveness study, which is likely the only feasible approach for evaluating the effectiveness of current EDPS, as a randomized controlled trial, by limiting access to EDPS for a large proportion of children would be unethical. As a result, our findings reflect real life trends and outcomes in an uncontrolled setting. Additionally, the severity of asthma differed between groups whereby 18.9% of patients in the EDPS group had mild asthma and 48% who did not receive EDPS also had mild asthma. This proportional difference may reflect the lower likelihood of physicians providing EDPS to a child presenting with mild asthma. Moreover, children with mild asthma presentations were less likely to return with a subsequent ED visits which may partially explain why there was not a significant difference in ED repeat visits. Though we undertook a sensitivity analysis to better understand the effectiveness of EDPS in children with milder versus more severe asthma presentations, we were limited by our small sample size and this approach may not be sufficient to be conclusive. Future studies with larger sample sizes in other settings may be needed to strengthen the evidence base. Although our study results should be interpreted with some caution given the unbalanced study sample, our findings are congruent with trends in repeat asthma ED visits seen at both at CHEO and across Canada over the past ten years, where the Canadian Institutes of Health Research [9] have shown that the rates of repeat asthma ED visits in children have not changed over the past decade. Therefore, despite some limitations, our study adds to the growing evidence-base which suggests that EDPS in Canada may not be enough and additional approaches to prevent repeat asthma ED visits should be explored.

### Present improvements and future directions

Since this study was carried out at CHEO, we have now implemented several improvements and have developed a targeted approach for offering specialized care for children at higher risk for repeat asthma ED visits. A comprehensive asthma program was launched in 2018 at CHEO which involved extended nurse and staff training, interviews examining current practice barriers and needs, the use of an electronic medical record (EMR) to streamline referrals for comprehensive asthma education directly from the ED, and provision of virtual asthma education for eligible patients [28]. Evaluation of this new program is currently under way.

## Conclusion

In summary, current emergency department strategies (EDPS) may be insufficient for preventing repeat asthma ED visits at one year, with one in four patients continuing to seek medical care at the ED setting. Current EDPS may need to be updated to meet the dynamic needs of patients from a real-world effectiveness and sufficiency perspective. While EDPS was offered in 87% of patients, these strategies should be revisited to optimize their implementation, particularly in patients at highest risk of repeat visits. We recommend stronger emergency department discharge strategies that may better target vulnerable children at highest risk such as patients with a history of previous ED visits and higher PRAM scores at triage, irrespective of whether they already have a primary care provider. We further propose that additional strategies should be considered, including referral to asthma specialists and/or comprehensive tailored, and multi-modal asthma education for children with specific risk factors for asthma ED re-visits. We propose that future work to leverage emerging smart technologies. For example, ED alert systems that integrate AI and machine learning methods into the clinical EMR may play an important future role in ascertaining patient risk profiles and enabling tailored EDPS for the most vulnerable patients as a way to improve asthma control and reduce the frequency of repeat asthma ED visits.

### Electronic Supplementary Material

Below is the link to the electronic supplementary material.

Supplementary Material 1
