# Supplementary material for: A real-world evaluation of the effectiveness and Sufficiency of Current Emergency Department Preventative Strategies for Reducing Emergency Department revisits in a Canadian children’s hospital: a retrospective cohort study
Source: Allergy Asthma Clin Immunol. 2024 Jun 25;20:37. doi: 10.1186/s13223-024-00900-z (PMC11197375; doi:10.1186/s13223-024-00900-z)
Supplement: Supplementary file 1 — Supplementary Material 1 [file 13223_2024_900_MOESM1_ESM.docx]

**Supplementary Tables**

Table S1. Summary of Asthma Emergency Department Discharge Strategies Adapted from The Canadian Pediatric Society Position Statement on Managing an Acute Asthma Exacerbation in Children (2021)(11) and Canadian Thoracic Society Asthma Consensus Guideline (2021)(12).

| **Patient** | | | **Inhaled Corticosteroids** | **Oral Corticosteroids** | **Action**  **Plan** | **Primary Care Follow-up** | **Specialist Referral** |  | |  | |
| --- | --- | --- | --- | --- | --- | --- | --- | --- | --- | --- | --- |
| Children and youth | Prescribe daily low dose ICS:  If 1. Asthma symptoms > 2 days/week, or has asthma related sleep disturbances (waking) > 2 x /month.  2. Moderate to Severe flares over the past year requiring oral corticosteroids  Increase to medium dose ICS if the child is not sufficiently responsive.  (For children/adolescents > 12 years, combination long-acting beta-antagonist-2/medium ICS dose can be used) | | | For any moderate-severe asthma flare**: Provide 1–5 days of oral corticosteroids  (Prednisone  Or dexamethasone) | Teach children and families how to use asthma inhalers and limit environmental triggers such as tobacco smoke, allergens, pollution | Follow-up with a primary care provider and/or asthma educator  by 4 weeks post ED  visit | Refer to Specialist if the child/adolescent has ≥ 2 flares on moderate dose ICS  Or any PICU admission | |  | |  |
|  | |  | |  |  |  |  |  |  |  |  |
| Pre-schoolers | | Prescribe daily low dose ICS if  1. Experiencing asthma symptoms > 8 days /month  2. Moderate to severe flares over the past year requiring oral steroids  Increase to medium* dose ICS if the child is not sufficiently responsive. | |  |  |  |  |  |  |  |  |

ICS = inhaled corticosteroids, ED = emergency department, PICU = pediatric intensive care unit

*Note that recommended dosing for low and medium dose inhaled steroids differs for preschool aged children (1–5 years old) compared to older children and adolescents

**Moderate to severe is based on a Pediatric Respiratory Assessment Measure (PRAM) of 4–12 at triage (18).

**Table S2**. Characteristics and exposures of individuals who presented with moderate to severe PRAM scores only.

|  | EDPS* (n = 755) | EDPS not documented (n = 109) | p-value |
| --- | --- | --- | --- |
| Age, *years* (mean, SD) | 4.7 (3.5)  [4.5–4.9] | 6.0 (4.6)  [5.3–6.7] | < 0.01 |
| Sex *Male (N, %)* | 513 (67.9%) | 71 (65.1%) | 0.56 |
| PRAM at index visit:  4–7 (moderate)  8–12 (severe) | 339 (44.9%)  416 (55.1%) | 51 (46.8%)  58 (53.2%) | 0.71 |
| Prior history of asthma | 563 (74.6%) | 82 (75.2%) | 0.56 |
| Allergy:  None  Nuts  Food  Other | 474 (62.3%)  137 (18.1%)  119 (15.8%)  145 (19.2%) | 63 (57.8%)  21 (19.2%)  19 (17.4%)  27 (24.8%) | 0.32  0.77  0.66  0.17 |
| History of exposure to tobacco smoke | 43 (5.7%) | 7 (6.4%) | 0.76 |
| Has a primary care physician | 651 (86.2%) | 94 (86.2%) | 0.99 |
| Prior ED visits | 135 (17.9%) | 19 (17.4%) | 0.91 |
| Return ED visit or hospitalization | 191 (25.3%) | 32 (29.3%) | 0.37 |

SD = standard deviation, 95% CI = 95% confidence interval, PRAM = pediatric respiratory assessment measure, EDPS = emergency department preventative strategies

*Definition of EDPS is having documentation of Asthma Action Plan (AAP) OR controller inhaler prescribed OR referral to specialist

**Allergies were divided into categories. Nuts = all nuts including peanuts and tree nuts; food = all foods excluding nuts; other = all reported allergies that were not to nuts or food types, e.g. pollen, dust, etc.

**Table S3.** Predictors of those who received EDPS (moderate to severe PRAM only).

| Odds Ratio Estimates | | | | |
| --- | --- | --- | --- | --- |
| Predictor | Odds ratio | 95% Confidence Interval | | p-value |
| Age | 0.97 | 0.91 | 1.03 | 0.26 |
| Sex (male vs. female) | 1.11 | 0.72 | 1.69 | 0.65 |
| PRAM (Severe vs. moderate)* | 1.02 | 0.67 | 1.55 | 0.92 |
| Prior diagnosis of asthma | 1.04 | 0.68 | 1.55 | 0.89 |
| Second hand smoke exposure | 0.88 | 0.38 | 2.03 | 0.76 |
| Primary care provider | 1.01 | 0.56 | 1.82 | 0.99 |
| Allergy: Nut vs. other allergies absent** | 1.04 | 0.57 | 1.89 | 0.89 |
| Allergy: Food vs. other allergies absent** | 0.90 | 0.49 | 1.66 | 0.73 |
| Allergy: Other vs. other allergies absent** | 0.81 | 0.48 | 1.34 | 0.41 |
| Prior ED visit within previous year | 1.07 | 0.62 | 1.86 | 0.82 |

PRAM = pediatric respiratory assessment measure, EDPS = emergency department preventative strategies. Definition of EDPS is having documentation of Asthma Action Plan (AAP) OR controller inhaler prescribed OR referral to specialist

* PRAM severity was defined as follows: Mild = 0–3; Moderate = 4–7; Severe = 8–12

**Allergies were defined as follows: Nuts = all nuts including peanuts and tree nuts; Food = all foods excluding nuts; Other = all reported allergies that were not to nuts or food types, e.g. pollen, dust, etc.

**Table S4**. Patient characteristics associated with odds of future acute asthma visits (moderate to severe PRAM only).

| Odds Ratio Estimates | | | | |
| --- | --- | --- | --- | --- |
| Predictor | Odds Ratio | 95% Confidence Interval | | p-value |
| Age | 0.95 | 0.90 | 1.00 | 0.04 |
| Sex (male vs. female) | 1.04 | 0.74 | 1.45 | 0.83 |
| PRAM score (severe vs. moderate) | 1.28 | 0.93 | 1.76 | 0.14 |
| Prior diagnosis of asthma | 1.57 | 1.05 | 2.34 | 0.03 |
| Second hand smoke exposure | 1.19 | 0.62 | 2.27 | 0.59 |
| Primary care provider | 2.12 | 1.24 | 3.61 | < 0.01 |
| Allergy: Nut vs. other allergies absent ** | 1.10 | 0.69 | 1.74 | 0.69 |
| Allergy: Food vs. other allergies absent ** | 1.24 | 0.78 | 1.98 | 0.37 |
| Allergy: Other vs. other allergies absent ** | 0.98 | 0.64 | 1.50 | 0.92 |
| Prior ED visit within previous year | 1.40 | 0.94 | 2.09 | 0.10 |
| Received EDPS | 0.79 | 0.50 | 1.24 | 0.30 |

PRAM = pediatric respiratory assessment measure, EDPS = emergency department preventative strategies. Definition of EDPS is having documentation of Asthma Action Plan (AAP) OR controller inhaler prescribed OR referral to specialist

* PRAM severity was defined as follows: Mild = 0–3; Moderate = 4–7; Severe = 8–12

**Allergies were defined as follows: Nuts = all nuts including peanuts and tree nuts; Food = all foods excluding nuts; Other = all reported allergies that were not to nuts or food types, e.g. pollen, dust, etc.
